# A Mechanistic Approach to Cross-Domain Perceptual Narrowing in the First Year of Life

**DOI:** 10.3390/brainsci4040613

**Published:** 2014-12-16

**Authors:** Hillary Hadley, Gwyneth C. Rost, Eswen Fava, Lisa S. Scott

**Affiliations:** 1 Department of Psychological and Brain Sciences, University of Massachusetts, 413 Tobin Hall/135 Hicks Way, Amherst, MA 01003, USA; E-Mails: hhadley@psych.umass.edu (H.H.); efava@psych.umass.edu (E.F.); 2 Department of Communication Disorders, University of Massachusetts, Amherst, MA 01003, USA; E-Mail: grost@umass.edu

**Keywords:** face perception, speech perception, word learning, perceptual narrowing, top-down, bottom-up

## Abstract

Language and face processing develop in similar ways during the first year of life. Early in the first year of life, infants demonstrate broad abilities for discriminating among faces and speech. These discrimination abilities then become tuned to frequently experienced groups of people or languages. This process of perceptual development occurs between approximately 6 and 12 months of age and is largely shaped by experience. However, the mechanisms underlying perceptual development during this time, and whether they are shared across domains, remain largely unknown. Here, we highlight research findings across domains and propose a top-down/bottom-up processing approach as a guide for future research. It is hypothesized that perceptual narrowing and tuning in development is the result of a shift from primarily bottom-up processing to a combination of bottom-up and top-down influences. In addition, we propose word learning as an important top-down factor that shapes tuning in both the speech and face domains, leading to similar observed developmental trajectories across modalities. Importantly, we suggest that perceptual narrowing/tuning is the result of multiple interacting factors and not explained by the development of a single mechanism.

## 1. Introduction

During the first year of life, infants begin to communicate and interact with the surrounding social world. Caregivers are important sources of social information and infants are particularly good at honing in on caregivers’ faces as well as their speech in the context of communication. The perceptual changes that occur during social learning in infancy have been referred to as “perceptual narrowing” or “perceptual tuning” and the developmental trajectory is similar across modalities. Specifically, perceptual narrowing refers to a decline in the ability to differentiate faces within infrequently experienced groups or speech within non-native languages, while perceptual tuning refers to the improvements in discrimination ability for frequently experienced groups of people or languages [1].

Perceptual narrowing has now been shown across several laboratories and in multiple modalities [2,3,4]. In Werker and Tees’ [2] seminal investigation, it was reported that 6-to-8-month-old monolingual English infants were as proficient as adult native Hindi speakers at discriminating non-native phonemic contrasts, such as the Hindi voiceless unaspirated retroflex *versus* dental place of articulation distinction (/ṭa/ and /ta/). In contrast, 10- to 12-month-old monolingual English infants failed to differentiate this non-native (/ṭa/ and /ta/) contrast. Similarly, Nelson [5,6] proposed that infants’ perception of faces also undergoes experience-dependent narrowing. The first empirical study to show perceptual narrowing for faces found that 6-month-old infants visually discriminated two different monkey faces as well as two different human faces. In contrast, 9-month-old infants and adults only showed evidence of discrimination for human faces [7]. Since these original investigations, a series of studies have been conducted suggesting that as infants approach one year of age, their discrimination of faces within infrequently encountered groups and phonemic contrasts within infrequently experienced languages declines relative to their discrimination of faces within a familiar group [7,8,9] or phonemic contrasts within their native language [2,10,11,12,13,14].

Although a robust effect, at this time, the mechanisms that underlie perceptual narrowing and tuning across domains are poorly understood. Here, we offer a top-down/bottom-up framework for examining perceptual narrowing and tuning for faces and speech and propose a specific top-down mechanism that may influence perceptual narrowing across domains. The benefit of this mechanistic approach is twofold. First, it is useful to consider the proximate underlying mechanisms of perceptual narrowing using mutliple methods and levels of analysis. This approach will help determine how and when learning (e.g., word learning, formation of categories or concepts, attention to social cues, improvements in communication skills, *etc.*) influences perceptual narrowing and tuning. Second, a mechanistic approach more readily allows for the formation of testable hypotheses, the result of which can further our understanding of infant learning and development.

The linked nature of visual and auditory systems has been systematically reported in studies investigating the development of multisensory perception. Similar to unimodal studies in the domains of language and vision, Lewkowicz and colleagues [15,16], (for a review, see [17,18]) report perceptual narrowing across the first year of life in experiments examining cross-modal matching of human and monkey faces and voices/calls. Additionally, infants’ cross-modal matching of speech tunes to the native language between 4.5 and 11 months of age, with narrowing for fluent speech [19] occuring earlier than for phonemes [20]. The parallel perceptual narrowing trajectories for face and speech perception, as well as multimodal perception within the first year of life resulted in proposals of a domain general mechanism underlying development across modalities [5,15,21]. However, whether or not one or more domain general mechanims exisit and the nature and specificity of such mechanism (s) are, at present, poorly understood.

Three recent reviews have begun to summarize and integrate research across the language and face domains. These reviews have synthesized the perceptual narrowing literature and have not only found numerous similarities but also important differences [1,22,23]. Maurer and Werker [1] compared perceptual narrowing for face and speech processing, with a focus on differences in timing, and the mechanisms (brain maturation and experience) that may be responsible for beginning the process of perceptual narrowing within and between domains. The authors specifically describe three important unexplored or underexplored questions in the area of perceptual narrowing. First, although the source of timing differences between narrowing trajectories for different components of speech (e.g., how salience of distinctions contributes to timing difference between tuning for consonants *vs.* vowels) has been explored, sources of timing differences between trajectories for different face groups (e.g., other-species *vs.* other-race) have not been investigated in depth (but see [24] for a recent discussion of timing in face tuning/narrowing). Second, Maurer and Werker [1] suggest that further work should investigate whether timing differences within and across domains reflect experiential differences or if they are guided by maturational constraints. Finally, the authors suggest that speech perception has a biologically-based sensitive period that is difficult to accelerate [25,26], while face perception may be more experientially-driven and malleability may depend on experience and/or lack of input [27]. However, the relative contributions of biological maturation and experience are difficult to disentangle in the domain of speech perception given the overlap between unique and specific brain development and speech exposure that occurs *in utero* [28]. Additionally, Maurer and Werker note that there has been a lack of comparable experiments across domains making these comparisons somewhat less tenable. This review provided a needed focus on the processes that initially drive perceptual narrowing.

Similar to Maurer and Werker [1], Flom [22] reviews the role of experience in altering the timing and flexibility of perceptual narrowing for speech, face, and intersensory/cross-modal perception. He argues that current methods used to study narrowing (e.g., behavioral habituation, electrophysiological recordings) provide contrasting results, such that observed behavioral findings do not always map on to neurophysiological findings. Further, he suggests that current methods fail to capture individual differences in narrowing trajectories and do not adequately explain its multifaceted nature. Flom proposes a re-conceptualization of perceptual narrowing with a focus on neural mechanisms to better investigate it across domains.

Finally, in a third review paper, Pascalis and colleagues [23] argue that perceptual narrowing is a phenomenon inherent to all systems involved in social communication (e.g., faces, speech, gestures, *etc.*). The authors suggest that the narrowing process leads infants to expertly process communicative information from important individuals such as caregivers. The authors propose that systems responsible for perception of social cues in different domains are interactive and linked and further suggest that infant narrowing occurs through a cross-modal mechanism specific to social cognition. In this regard, perceptual narrowing can be viewed as the result of interactions between systems used for processing faces and speech with the goal of social communication.

Altogether, these recent reviews have framed current discussions regarding the developmental trajectory and nature of perceptual narrowing across domains. Here, we propose a framework that we hope will aid future investigations and identify specific shared mechanisms that drive perceptual narrowing. Maurer and Werker [1] discuss mechanisms that may trigger the onset and offset of tuning, and make excellent points about future research investigating the reasons for timing and malleability differences between domains. Similarly, Pascalis [23] proposes a general perceptual narrowing process inherent to all social domains. Flom’s [22] review of the role of experience in the timing and flexibility of narrowing gives rise to a still-open question of how experience might modulate aspects of tuning. Through his discussion of methodological challenges, Flom also highlights the need for converging methods (e.g., behavioral and neural) in the study of perceptual narrowing. Indeed, as will be discussed below, some of the questions Flom poses have already been addressed in recent empirical work within (but not across) domains. Discussion of the neural mechanism (s) that underlie perceptual narrowing is a potentially critical piece in determining the extent to which narrowing/tuning is linked across domains, or whether narrowing/tuning is achieved via different mechanisms that result in similar outcomes. Here, we argue that cross-domain and multi-method experimental research, with an eye toward understanding process, mechanism, and outcome, should be a major focus of future investigations in perceptual narrowing and we offer a top-down/bottom-up framework as a guide.

Top-down processing involves recruitment of higher-level information and/or knowledge (e.g., conceptual knowledge, prior experience, *etc.*), which influences perception and processing [29,30,31]. One example of how top-down processing operates is through selective attention. In top-down processing, attention is guided towards task-relevant locations and/or features [30,31], and can also influence perception through expectation and task context [30]. In contrast, bottom-up processing refers to processing that is driven exclusively by external stimuli and their perceptual features [29,31]. Bottom-up processing is typically related to sensory salience of stimuli, and activates exogenous attention [31]. In this paper, we discuss both exogenous (*i.e*., environmental or stimulus driven) and endogenous (*i.e*., voluntarily controlled) attention and their relation to bottom-up and top-down influences. We focus our review on the development of perceptual narrowing as it relates to higher-level perception of faces and speech as well as how developmental mechanisms, including endogenously-controlled selective attention, word learning, and category and concept formation, influence perceptual development during the first year of life. We will discuss evidence demonstrating that as speech and face perception develop, they are guided first by bottom-up learning and then a combination of bottom-up and top-down information. In addition, we specifically propose word learning as a particularly important top-down factor shaping face and speech perception.

## 2. Bottom-Up Influences

Speech is a complex stimulus. Infants eventually glean meaning from this rapid and intricate audiovisual signal that is tied to the interactions they share with their caregivers. Ultimately, *in*- and *ex-utero* experience is a balance of noting the many components of the speech signal that infants can perceive and those that are important for meaningful distinctions in their native language. The acoustic properties of the speech stream interact with biological maturation beginning *in utero*. This is possible because the auditory system is sufficiently formed for fetuses to begin hearing at least some of the input in their environment by 19 weeks gestation, with all sound below 1000 HZ available to most by 26 weeks gestation, and the full range of human hearing by 35 weeks gestation [28]. Fetal EEG readings confirm significant neuroplasticity and reorganization during this period (see [32] for a review).

Speech processing *in utero* further predicts the acoustic properties (*i.e*., components of the speech signal) to which newborns respond. This is reflected in both behavioral (e.g., [33,34]) and EEG data [35]. For example, newborns do not appear to distinguish between languages with a prosodic structure similar to that of their native language (e.g., English, Dutch) [33,35], (see also [36]). Prosodic patterns are carried on low frequencies, which can be conveyed to the fetus in the womb, resulting in in utero experience with this component of language [28]. Thus, newborns’ perception of the speech signal is initially shaped by an accumulation of *in utero* perceptual experience.

Infants continue to build upon their *in-utero* experience and add the use of other cues (e.g., phonemic and phonological cues) at different levels of the speech signal. By incorporating the use of additional speech cues with experience and biological maturation, by 5 months, infants can differentiate between languages within the same rhythmic class (*i.e*., tone discrimination [37]). Ultimately, infants narrow their understanding and perception to the components of speech salient in their native tongue relative to other languages. This narrowing has been traditionally discussed at the phonemic level. For example, the /li/ portion of word “lip” can be produced in slightly different locations within the mouth (e.g., the tongue being placed behind the teeth or curled back on the palate), and this production difference can be perceived. However, depending upon the language, these production “types” are collapsed across (English) or maintained as meaningful differences (Hindi) (see [38,39] for a classic example).

Taken together, the reviewed findings related to early preference and discrimination [28,32,36,37,39,40,41] are remarkable given how rapidly speech is produced and therefore must be perceived. Indeed, this property of the acoustic speech signal might be one further source of bottom-up information that infants can use to improve their processing and understanding. For example, according to Cutler and Mehler’s *periodicity bias* [42], (see [43] for a review), sustained acoustic information (e.g., a vowel) causes a speech sound to be narrowed to a native-language category earlier than more punctual, discrete acoustic information (e.g., a consonant). In addition to showing another example of how infants use bottom-up information during perceptual narrowing, this hypothesis could explain infants’ earlier responses to phonemes that contain both low-frequency information and are sustained, or have comparatively longer duration. For example, vowel perception narrows by 6 months [14], and perception of nasal contrasts narrows by 6 or 10 months, depending on salient qualities of the contrast other than length [44]. However, duration in time is not the only feature of the acoustic signal that makes a category salient. For example, fricatives such as /s/ and /f/, which rely on high-pitched information and are discriminable by the combination of multiple cues (see [40] for a discussion), may be learned later despite their length [41,45]. As a general statement, the perception of native phonetic categories has typically narrowed by 12 months of age in a monolingual infant [2]. But this process continues to unfold after the first year of life, as children do not yet fully respond to native-language /r/ *vs.* /w/ cues as late as five years [46], again, despite their length.

The dominant model for bottom-up learning of phoneme contrasts has been distributional learning (see [47] for a thorough review). Maye, Werker, and Gerken’s [48] proposed that infants carve out phonemic representations from their experience. Specifically, Maye and colleagues created a continuum of speech tokens between an unaspirated voiceless alveolar stop [t] to a voiced alveolar stop [d], and presented one of two distributions of these tokens to 6 month-old infants. Infants who were trained on a unimodal distribution, where the majority of exemplars heard were almost a perfect “mix” of [t] and [d] at the center of the continuum did not discriminate between two endpoint tokens at test. In contrast, infants who were trained on a bimodal distribution, where the majority of exemplars were taken from near two endpoints of the distribution (e.g., half of their exemplars sounded almost like a “perfect” [t] and the other half sounded almost like a “perfect” [d]) did discriminate between the two endpoints at test. Hearing two clusters of tokens led to infants perceiving two phonemes, while hearing only one lead to the same tokens being perceived as identical (see also [49] for a replication). Similar experiments with 10-months-olds demonstrate that this ability continues through the period when children show perceptual narrowing for the contrast [50]. This suggests that perceptual narrowing is not driving this ability, but instead, infants appear to shape their perception of phonemes and respond to the bottom-up cues of variation/distributional information in the speech they hear.

Another model for the development of speech perception is a four-stage model proposed by Kuhl [51] that includes both bottom-up and top-down mechanisms. In this model, infants move from an initial state of broad phoneme discrimination ability to a final state where they have developed neural systems committed to preferentially processing native-language phoneme distributions. Bottom-up influences are suggested to shape early discrimination abilities and continue to play a role later in the first year, after perceptual narrowing has occurred. Initially, infants discriminate equally well across native and non-native speech sounds, and performance can be affected by low-level acoustic salience. As infants continue to gain experience with language, information is made salient via exaggerated cues presented in infant directed speech. Finally, even after infants demonstrate narrowing to native phoneme contrasts, phonemic discrimination can be elicited through changing distributional patterns. Therefore, according to Kuhl’s model, bottom-up influences (e.g., acoustic salience) exert a strong influence early in development on phonemic discrimination. Use of phonological cues continues (alongside top-down influences, discussed in the next section) post-narrowing.

In contrast to language (with which infants receive prenatal exposure), experience with faces begins at birth (although see [52,53] for proposed *in utero* proprioceptive face experience). Newborns and young infants show a visual preference for faces over other types of objects and patterns [54,55]. Interestingly, this preference is apparent even when stimuli are perceptually equated and the only difference is whether inner elements are arranged in a face-like (top-heavy) or non-face-like (bottom-heavy) way [54]. However, the underlying nature of this preference, and whether it is driven by low-level stimulus properties or is face specific, has been a subject of much debate [5,56,57,58,59,60]. Evidence suggesting that infant’s initial preference for faces over objects may be driven by low-level perceptual properties comes from work that finds a general preference for ‘top-heavy’ stimuli in newborns that may explain early face preferences [56,57,58]. This top-heavy preference has been suggested to be a result of an upper visual field bias, leading to preferences for top-heavy configurations [57]. Although newborns show a preference for faces or face-like stimuli, they do not show a spontaneous visual preference for own- *versus* other-race faces [61]. Newborns also fail to show a visual preference for their mother’s face over a stranger’s face without prior experience hearing their mother’s voice. However, newborns do prefer to look at their mother’s face after viewing her face paired with her voice immediately after birth, suggesting very fast and early learning, associated with linking the mother’s voice with her face [62]. A domain-general preference for top-heavy stimuli (including faces) at birth paired with a lack of discrimination or preferences for specific types of faces prior to experience suggests that face preference at birth is likely driven by bottom-up mechanisms.

Although not present in newborns, 3-month-old infants exhibit a visual preference for own- *versus* other race faces [9,61], as well as a preference for human relative to monkey faces [63,64]. No own-race preference is found when infants are raised in predominantly other-race environments [65]. Three-month-olds also fail to differentiate other-race faces when habituated to a single face, but successfully discriminate among other-race individuals following habituation to multiple faces [66]. Taken together, these results indicate simple perceptual experience with a face group may influence face processing within the first few months of life. This perceptual experience may also impact the pattern of narrowing that occurs in the first year [7,8,9]. Infants begin by exhibiting discrimination for faces within multiple groups at 3-months, and then only show discrimination for own-race groups and groups that are thought to be more similar to the own-race group (e.g., Caucasian 6-month-olds discriminate Asian faces, but not African-American faces). Finally, by 9-months of age, infants only show discrimination of own-race faces [8,9]. These findings suggest that tuning is gradual and may be influenced in part by the perceptual similarity of unfamiliar face groups to the race/group (or species [7,67]) with which one has the most experience.

Researchers who aim to understand the development of face biases in the first year of life often turn to perceptual and social theoretical frameworks within the adult literature (for review, see [68]). One persisting model of adult face encoding and recognition that highlights the importance of bottom-up contributions in adult face biases is the Multidimensional Space (“face space”) framework [69]. This framework posits that faces are encoded in a multidimensional space, vary on different dimensions, and surround an averaged prototype. This framework suggests that an individual’s face space is created through experience with face exemplars, akin to distributional and/or statistical learning. The face space model has been applied to the progression of perceptual tuning/narrowing in infancy as well. Kelly and colleagues [9] argue that infants build their face space around same-race faces, resulting in the observed decline in discrimination and recognition ability for other-race faces by 9 to 12 months of age. Therefore, accumulation of visual perceptual experience (*i.e*., simple exposure) to a face group may act as a bottom-up influence in shaping perceptual narrowing. Here, the quantity and not the quality of interactions is thought to be the mediating factor.

A recent study by Balas and colleagues [70] provides further evidence that older infants may still use bottom-up information when processing faces. Nine-month-old Caucasian infants viewed computer generated faces while ERPs were recorded. Faces were either own- (White) or other-race (Black) faces across dimensions of face shape and pigmentation, creating four face types: (1) White shape-White pigmentation, (2) Black shape-Black pigmentation, (3) White shape-Black pigmentation, and (4) Black shape-White pigmentation. Infants exhibited a larger neural response to “realistic” same-race faces (White shape-White pigmentation) relative to ‘realistic’ other-race faces (Black shape-Black pigmentation). This differential response was not present for ‘hybrid’ faces, indicating that both face shape and pigmentation information influence face processing at the neural level. These findings suggest that at 9 months, bottom-up information, including both shape and pigmentation, influence the brains response to faces.

In summary, speech and face perception in the first few months of life are heavily impacted by bottom-up factors including stimulus salience (e.g., acoustic salience and top-heavy distribution) and amount of perceptual exposure. In particular, as infants are exposed to speech and faces, statistically-based distributional cues shape discrimination ability [48,50,65]. Later in infancy (even after perceptual narrowing has occurred), these bottom-up factors continue to influence phoneme and face perception, but are joined by top-down influences.

## 3. Top-Down Influences

Although it is evident that bottom-up factors play a role in shaping perceptual narrowing in both speech and face domains, there is ample evidence to suggest top-down factors also influence narrowing during the first year of life. With respect to speech perception, we review findings highlighting the importance of social context and word learning. Within the face domain, we discuss influences of socio-cultural context, learned attention, category and concept formation, and—parallel to the speech domain—word learning.

Kuhl’s [51] four-stage model of speech, discussed in the previous section, also includes top-down influences on the development of speech perception. Following the initial stage of broad discriminatory abilities driven by perceptual input and acoustic salience, in the second stage, infants’ speech and phoneme perception is impacted by social interactions with adults. The social context itself (e.g., live, partner-contingent interactions), rather than the perceptual experience that arises from such interactions, might act as a top-down factor in shaping tuning for phoneme discrimination ability [71]. Additionally, Kuhl also suggests in the third stage of the model that word learning and word knowledge may help sharpen native phoneme distinctions. Evidence for both social context as well as word learning as top-down influences will be discussed in the following paragraphs.

Kuhl and colleagues have argued that social context operates above and beyond perceptual experience to shape perceptual tuning to native phoneme contrasts [71,72]. In one of the few longitudinal studies exploring perceptual narrowing for phoneme perception, 9-month-old American infants were exposed to Mandarin 12 times over a 4–5 week period. Following this exposure, infants responded similarly to Taiwanese-learning (monolingual) infants on a Mandarin phoneme contrast. A control group of English-learning infants not exposed to the Mandarin speaker did not respond to the Mandarin phoneme contrast [71]. Importantly, groups of American English-learning infants exposed to Mandarin via taped audiovisual presentations responded similarly to the English-only group, leading Kuhl and colleagues to argue that infants rely on the social in-person interaction with another person to maintain discrimination between speech sounds that are within the same category (and therefore not meaningful) in the native language. That is, only in the presence of live human contact and contingent interactions do infants override the statistical disadvantage of their experience and instead perceive a meaningful distinction between two speech sounds they had previously collapsed across. Kuhl therefore argues that development in speech perception is driven by top-down social learning mechanisms [72].

The dominant model in perception of phonemes in older children and adults has been a lexical one: that is, our existing mental dictionary and the frequency with which we use its contents dictate how easily we process speech sounds (see [73] for a classic example). The lexicon acts as a top-down organizing mechanism for perception of speech in both adults [74] and infants [75]. That lexical activation processes underlie basic speech perception is unsurprising given that the phoneme has been defined lexically, as the smallest unit of acoustic or articulatory change that yields a difference in words. Recent work in infant word learning has demonstrated that infants might have lexical representations for a small number of words as early as 6 months [76]. Bergelson and Swingley [76] tested 6–9-month-old infants on familiar words in a language-guided test to examine how infants responded to words embedded in a thematic sequence. Although 6-month-olds had previously been reported not to respond to a word like “apple”, when the testing image is an isolated apple [77], they did respond when the word “apple” was paired with a picture of a face eating the apple. This is not a strict demonstration that 6-month-olds know the isolated meaning of “apple” but instead suggests that they have begun developing semantic networks for the word. Importantly, though, nascent semantic networks might be all that infants need in order to use *words* to bootstrap phoneme perception.

Baysian models can use top-down as well as bottom-up mechanisms to learn phonemes [78] with very little semantic information attached to a word form—even something as simple as where the word (*i.e*., context) was spoken (which should be fairly salient information: [79]). Moreover, both adults and 8-month-old infants trained on a non-native phonemic contrast in the context of contrasting words discriminated that contrast, while those trained on non-contrasting words did not [80]. This paradigm was similar to the one used by Maye and colleagues [48,49] described previously: participants were trained on 64 word exemplars where the relevant contrast was either contrastive or non-contrastive. The difference in this case is that it was the words, rather than distributional properties of the phonemes, that differed (see [81] for a similar demonstration in 14-month-olds). Eight-month-olds demonstrated phonetic learning as predicted by the Baysian models: they learned and generalized speech sounds when words—with loose meanings—were associated to the phonemes. Feldman and her colleagues have therefore argued that infants might use both the phonotactic and semantic properties of words to perceive speech. Similarly, English-learning 9-month-olds show heightened sensitivity to a Cantonese-specific phoneme contrast when trained as words; that is, when they are paired with referents [82]. This is not only the case for phoneme perception, but speech perception of prosodic patterning is also affected when words with referents are trained [83]. Following this logic, much of the work on early word perception suggests that the development of word meaning guides—or retards—perceptual narrowing for phoneme perception. As infants learn words with distinct meanings, they will better detect differences between phonemes that distinguish words and become worse at distinguishing phonemes that do not differentiate native words.

Within the domain of face perception, adult-focused social cognitive frameworks provide strong evidence that face biases in adulthood are not entirely the result of basic perceptual input and exposure (e.g., [84,85,86]). One particular top-down cue that has been considered in the adult literature is the influence of culture on face perception. For example, using eye-tracking, Asian adults demonstrate concentrated looking at central face regions while Caucasian adults fixate mostly on the eyes and mouth [87]. Furthermore, these differential scan patterns may be shaped by early social experience. Kelly and colleagues [88] examined British-born Chinese adults’ scan patterns and behavioral discrimination of Caucasian and Chinese faces. Findings indicated that the majority of the British-born Chinese adults tested in the study showed “Eastern” gaze patterns (concentrated looking at central features) for both Caucasian and Chinese faces, although a subset demonstrated “Western” gaze patterns (looking at the eye and mouth regions). Interestingly, although gaze fixation patterns differed there were no differences for behavioral measures of discrimination. The authors suggest that even though British-born Chinese adults received multiple years of experience with Caucasian faces, early culture-specific social interactions with caregivers strongly influences gaze patterns. Differential gaze patterns are arguably related to selective attention (e.g., attention to parts of the face that convey relevant information), however this does not preclude them from being influenced by culture. That is, cultural values (e.g., not engaging in direct eye contact), a top-down mechanism, could impact the development of selective attention to face regions. It should be noted that morphological differences in facial features have been measured for faces of different races, indicating the possibility that differential scanning patterns may be due to bottom-up stimulus features [89]. However, the findings by Kelly and colleagues [88] suggest that morphological differences cannot fully explain differences in effective gaze patterns, as both “Western” and “Eastern” gaze patterns led to successful behavioral discrimination of Caucasian and Chinese faces.

Within the infant literature, recent eye-tracking studies suggest that socio-cultural norms may also impact how older infants visually scan own- and other-race faces. Between 4 and 9 months of age, native Chinese infants decrease looking to internal features—particularly the nose—of other-race, but not own-race faces [90]. Similarly, between 6 and 10 months, Caucasian infants increase looking to the eye region of own- but not other-race faces [91], and exhibit more frequent shifts between the eyes of own—relative to other—race faces [92]. These findings suggest that, similar to what is seen in adults, infant face scanning strategies are influenced by top-down, cultural factors. However, there is currently no evidence to suggest that face processing/recognition strategies, during development, differ across cultures. More specifically, it is currently unclear whether changes in face scanning patterns that occur during the same period as perceptual narrowing are the result of a cultural influence or whether they are simply based on perceptual experience with certain face groups—an influence that does not differ across cultures. The presence of cross-race morphological differences has also been noted in the infant literature, and has been incorporated into discussions of how scanning patterns may be influenced by contributions of the stimulus itself as well as the infant observer [90,91]. This point of view falls directly in line with the proposed bottom-up/top-down processing framework, whereby older infants make use of both bottom-up and top-down information. Future infant research should aim to sort out if and how cultural norms shape infants’ face scanning, and when or if these top-down culture-specific influences impact face discrimination or recognition.

Experience with certain face groups may also function to guide infants’ attention to relevant facial features. Simpson and colleagues [93] applied a learned attention model to the study of species-related tuning and examined human, monkey, and sheep face discrimination in young infants (4–6 months), older infants (9–12 months), and adults. They reported an increase in the number of facial features that can be discriminated, as well as an increase in how many species can be differentiated at 9–12 months relative to 4–6 months. Additionally, the types of features used to discriminate primate (monkey and human) faces were different than those used to discriminate non-primate species (sheep). The authors suggest that changing goals across development (e.g., older infants may look at regions that convey emotion) may guide infant looking to specific features necessary for extracting relevant information, indicating the presence of top-down learning in face perception.

In a similar vein, scanning of human faces is also likely impacted by changing developmental goals that operate at the level of selective attention. Between 4 and 8 months of age, infants shift their attention from the eyes to the mouth region of talking faces (for both native and non-native speakers). Around the same time, infants are also typically beginning to understand the meaning of familiar words [76], and produce speech-like sounds [94,95]. The parallel timing for the shift in scanning pattern indicates that as infants are beginning to learn words and produce speech, they are attending to adults’ mouths, particularly when adults are speaking [96]. Therefore, word learning and speech production are likely top-down influences in shaping face-scanning strategies.

A recent computational model for face tuning [97] suggests that the amount of perceptual exposure (functioning as a bottom-up factor) older infants have with a face group is not sufficient to explain behavioral perceptual narrowing (*i.e.*, failure to differentiate two other-race faces). Using a Bayesian approach that included perceptual information (number of face exemplars exposed to) as well as conceptual information (presence of race category boundaries), Balas accurately modeled perceptual narrowing to own-race faces as seen in infants. The results of the model suggest that although perceptual experience/input helps shape face discrimination ability, perceptual narrowing also relies on top-down processing wherein infants group faces according to norm-based and conceptually driven race categories. Experimental work has also revealed the presence of race-based face categorization in 9-month-old infants [98]. The authors suggest that these categories are in part a result of conceptual categorization, as 9-month-olds formed discrete categories for own-race and other-race faces (e.g., an own-race category that excludes other-race faces). The authors argue that in order to form two discrete categories, infants must override the spontaneous preference for own-race faces, suggesting that these categories are not purely perceptual in nature. These findings further indicate that infants may be representing face groups at a conceptual level.

A recent study examining the behavioral and electrophysiological correlates of face-related perceptual narrowing also demonstrates the influence of top-down conceptual information on face perception. Vogel, Monesson, and Scott [99] investigated the impact of race on how 5- and 9-month-old infants match emotion information for voices and faces. Consistent with previous reports, 5-month-old infants tell apart faces from both races, as indexed by novelty preferences after a familiarization period. However 9-month-old infants only differentiated between two own-race faces. In line with behavioral findings, electrophysiological results revealed race-specific perceptual processing of emotion-related face stimuli at 9 months. Specifically, 9-month-old infants differentiated emotionally congruent and incongruent face-voice pairs for own, but not other-race faces. Five-month-olds, on the other hand, differentiated emotionally congruent and incongruent face-voice pairs regardless of race. Due to the cross-modal nature of the stimuli used in this study (*i.e.*, congruency can only be detected across auditory and visual information), it can be concluded that infants draw on prior, top-down, knowledge of emotion processing in order to differentiate between emotionally congruent and incongruent face-voice pairs. Interestingly, neural responses related to detection of congruency were seen for an ERP component related to endogenously-driven selective attention in 5-month-olds, and for components related to perceptual processing in 9-month-olds (for review, see [100]). These findings suggest that younger and older infants utilize different neural mechanisms when completing this emotion congruency task.

Word learning, discussed earlier as a top-down influence on perceptual narrowing of speech perception, also appears to guide face discrimination abilities. Between 6 and 9 months of age, infants were trained to associate labels with monkey faces [101,102,103]. Infants who were trained with individual-level labels (e.g., “Fiona”, “Boris”) continued to discriminate monkey faces at 9 months of age whereas training with a generic, category-level label (e.g., all faces labeled “Monkey”) or exposure to faces without labels led to perceptual narrowing [103]. In order to understand the neural bases of perceptual narrowing, the Scott and Monesson also recorded also ERPs while infants viewed upright and inverted monkey faces before and after training [103]. Although holistic processing is multifaceted, a robust marker of holistic face processing in adults is the face inversion effect, or impaired recognition, delayed responses, and differential neural activity, for upside-down relative to upright faces [104,105,106]. In Scott and Monesson’s [103] study, individual, but not category or exposure training, led to a greater inversion effect and thus more specialized, holistic, and face-like (as opposed to object-like) neural responses for monkey faces. These results suggest that specialized neural representations for faces develop over the first year of life and are the result of experience learning to match faces with unique labels.

The lack of discrimination and expert neural processing following category-level and exposure training indicates that the amount of perceptual exposure alone (*i.e*., what infants would gain through strict bottom-up processing) is not sufficient to explain perceptual narrowing for faces. It is possible that the presence of unique speech referents (labels) aids in mapping distinct meanings onto individual monkey faces, prompting infants to attend to visual differences among the faces. In contrast, when a single, common speech referent (“monkey”) is applied to all faces, visual differences between face exemplars may not be meaningful or important for infants to distinguish.

Similar effects have been seen following training with other-race faces, either presented in picture books [107] or short videos [108]. In both studies, faces were paired with individual labels (in the videos, actresses named themselves). Following training, infants successfully discriminated other-race faces. It is possible that similar to Scott and Monesson’s [102,103] training study, word learning associated with book training [109] led infants to focus on meaningful differences between faces. Although unique labels were not paired as frequently with faces in the video training [108], a comparable conceptual link may have been made between unique voice and face identities (e.g., infants used cross-modal face and voice cues to create unique ‘person’ concepts).

A recent follow-up of Scott and Monesson’s [102,103] training study examined lasting effects of early labeling experience by assessing behavioral discrimination and neural processing for the trained category (monkey faces) and for human faces 3–4 years later [110]. Children, trained with monkey faces as infants, did not show enhanced behavioral or neural processing for monkey faces. However, compared to the category-level training group and a control group of children with no experience with monkey faces, children who received individual-level training as infants exhibited faster reaction times and more adult-like neural processing of human faces. These results indicate that in the absence of continued experience with a particular stimulus category (monkey faces), early experience with individual-level learning provides a later benefit for frequently encountered face groups (human faces). The authors discuss these findings in terms of *stimulus-specific* (learning is specific to an experienced stimulus or category) and *process-specific* learning (learning is not specific to the stimulus, but to the process of matching an individual-level name with a specific face), and conclude that early infant labeling experience primarily facilitates *process-specific* learning. The presence of generalizeable training effects provides evidence that infants learned a skill related to individuating exemplars and applied that skill to relevant categories (human faces). This early learning had lasting *process-specific* effects that resulted in a more general cognitive skill. Future research in this area needs to more clearly delineate the reasons for these lasting effects (e.g., possible role of the quality of parent-infant interactions or changes in the language environment in the home) and the extent to which learning generalizes across domains.

In sum, by approximately 5 or 6 months, top-down influences shape perceptual narrowing for speech and faces. In particular, social interaction appears to be one important factor that may facilitate discrimination of phonemic contrasts [71,72] and face processing [88]. Early social experience may also shape gaze patterns for face groups. However, it is unclear whether the same aspects of social interactions (e.g., live interaction, contingency) that have been found to be important for speech perception are also important for face perception. In addition, there is evidence that concept formation and specifically word learning act as a top-down factor in driving perceptual narrowing/tuning in both speech and face domains.

## 4. Word Learning as a Mechanism

In addition to demonstrating how a top-down/bottom-up framework is useful for characterizing the development of perceptual narrowing/tuning in speech and face domains, we also propose word learning as an influential top-down mechanism working to shape development across domains. With typical experience and development, infants respond to familiar words by 6 months of age [76], and are clearly quite good at it by 9–11 months [111]. *Word learning* here is defined as the association in memory of an auditory form—a series of speech sounds, such as /bɔrɪs/ (“Boris”)—and a semantic meaning—perhaps as simple as a visual referent, such as a picture of a monkey. However, learning a word does not necessarily involve representing its acoustic contents as separate phonemes. Rather, the acoustic contents of a learned word are conceptualized holistically (rather than as a sum of its individual phoneme parts).

Assigning meaning to a word can influence the perception of its individual phonemes in a top-down fashion. For example, when presented with novel words (e.g., “bih” and “dih”), 14-month-old infants do not differentiate the minimal pair (*i.e.*, /b-d/) [112], but when words are very well known (e.g., “ball” and “doll”), infants discriminate the minimal pair (*i.e.*, the /b-d/ contrast) [113]. Importantly, when a familiar word is paired with a mispronunciation or novel minimal pair (e.g., “baby” and “vaby”), infants will treat the mispronunciation as an instance of the known word [114,115]. That is, infants and young toddlers respond to a sparse underlying lexical representation, rather than robust phoneme-by-phoneme representations. Typically, in the second and third years of life, children come to represent the phoneme sequences in words in more robust, adult-like ways [116]. Furthermore, experience with words in general, as opposed to knowing specific words, shapes perceptual tuning to the particular phonemic contrasts present in the native language [117]. Nonetheless, knowing a word, or having a top-down conceptualization of meaning paired with phonological form, however sparse those representations might be, can facilitate infants’ treatment of a particular phonemic contrast over and above using bottom-up perception of the same contrast (bottom-up perception of the salient categories of speech sounds).

We argue that as infants form links between visual and speech referents, the two support one another. Word learning can also cross-modally facilitate visual perceptual narrowing/tuning, such that individual labels make perceptual differences between exemplars more salient, while category labels draw attention to common perceptual features. This pattern of effects has been demonstrated via eye-tracking [118,119]. When novel objects are paired with a shared label, infants direct their gaze to common features [118], and when objects are paired with individual labels, infants direct their attention to unique features [119]. It is possible that a similar process may have occurred in previous infant face-label training studies [102,103]. For example, correlating one monkey with the label /bɔrɪs/ (Boris) and another with the label /fionə/ (Fiona) sharpened infants’ perception of visual differences between the two monkeys. However, for infants who saw two monkey pictures, each labeled “monkey,” visual differences between them may be meaningless or unimportant. This is consistent with the hypothesis that young children’s lexical representations are de facto at the category level (*i.e*., “monkey”) unless specifically trained [120]. Therefore, we argue that top-down processing associated with word learning may help shape particular behavioral and neural responses to the referenced stimuli.

Converging evidence for this hypothesis comes from the literature on audio-visual speech perception. By 4½ months infants become susceptible to the McGurk effect [121,122], and by 5 months infants have become attuned to the mouth shape most typically associated with specific articulations [123]. However, the strength of this association (between mouth shape and speech sound production) seems to rely on the development of speech production. Children with poor articulation do not benefit from visual input in auditory perception as much as those with good articulation or adults [124]. Following this logic, Lewkowicz and colleagues proposed that the need to refine speech categories drives infant attention to the mouth [96]. As infants begin to produce canonical phonemes and syllables, their visual attention shifts toward the mouths of speaking adults [96]. In addition, the correspondence between audiovisual facial movement and speech output narrows to native-language categories as in unimodal perceptual narrowing [20]. Though no word meaning is overtly attached to the speech signal in these cases, infants may shift their focus to the mouth region of a face during the period of early word learning to disambiguate phonemes from one another. One benefit of this strategy would be to provide more robust representations of the phoneme sequences in words. Future work that examines infants’ learning of words that differ by a minimal pair in an audiovisual paradigm might help resolve this ambiguity, and might further elucidate the importance of social context in word learning. In addition, the visual shift in attention from the eyes to the mouth during word learning, as reported by Lewkowicz [96], may facilitate the development of holistic face processing. An investigation examining the relation between word learning (or language abilities in general) and holistic face perception might yield interesting connections across domains.

Finally, Kuhl’s [72] social gating theory posits that social context (e.g., live, contingent interactions) is necessary to facilitate learning of non-native speech contrasts in infancy. Nine-month-old infants discriminate non-native (Mandarin Chinese) phonemes following conceptually-based, social experience, but fail to make non-native discriminations following experience via an audio or audio-visual recording [71], suggesting that a social context may be necessary for word learning to result in differentiating non-native phonemes. It is less clear whether a social context is crucial for word learning to shape discrimination of visual categories. When infants are trained to match individual labels with unfamiliar faces (other-species or other-race) by taking home picture books, they successfully discriminate the faces at 9 months of age [102,103,109]. These book-training studies are social in nature, requiring parents to verbally label book images in an interactive way. Therefore, similar to phoneme perception, socially-based training results in discrimination of previously unfamiliar faces. However, daily video experience with other-race faces also led to discrimination of the face group in 8–10-month-olds, suggesting that live social interaction may not be necessary for infants to distinguish faces within other-race groups [108]. As discussed in the Top Down Influences section, the link between word learning and individuation of other-race faces in the video training study by Anzures and colleagues (2012) is not as clear as in book training studies [102,103,107], making it difficult to draw strong conclusions. Future investigations should continue to explore the role of social context in how word learning shapes visual discrimination of faces. Nonetheless, the inclusion of a social context (e.g., interactive experience with parents) for face labeling is representative of how word learning may function in everyday settings. Parents label frequently encountered faces, infants learn to associate names with these faces, and may also attend to differences in order to distinguish individuals. Moreover, following Lewkowicz’s proposal, social interactions between infants and parents during word learning may facilitate attention to the face and promote holistic perceptual processing. However, it is also currently unclear whether word learning can influence face perception in the absence of an interactive audiovisual social context.

In summary, we propose word learning as a top-down mechanism to facilitate perception of face and speech categories by directing attention to meaningful *versus* non-meaningful distinctions between phonemes or between visual stimuli (e.g., objects or faces). In the first year of life, caregiver labeling may be simultaneously influencing language development and face processing and result in the refining of visual and auditory systems such that narrowing and tuning effects are observed across domains.

## 5. Conclusion

Recently, there has been a great deal of progress in integrating perceptual narrowing/tuning research across speech and face domains, as well as in intersensory perception [1,23]. Here, we explore the mechanisms driving narrowing/tuning for faces and speech and propose the use of a top-down/bottom-up processing framework for understanding cross-domain similarities and mediating mechanisms.

Young infants appear to make use of bottom-up cues to process speech and face stimuli (speech: [48], faces: [66]) resulting in discrimination that can be manipulated by changing perceptual input (e.g., adjusting the distributional properties of phonemes or increasing exposure to face exemplars). By 5 months of age, top-down cues such as social context and word learning influence perceptual narrowing in both speech and face domains. However, bottom-up factors (e.g., amount of perceptual exposure) continue to influence speech and face discrimination abilities. Therefore, it appears that across both domains, infants transition from primarily relying on bottom-up cues to making use of bottom-up and top-down information.

We also propose word learning as a specific top-down mechanism that is, in part, responsible for narrowing/tuning across speech and face domains. Word learning bootstraps perception of social categories such as speech and face groups at a mechanistic level by creating meaningful distinctions both at the phonemic level of speech, and within face processing. In everyday life, word learning occurs as caregivers interact with their infants and label faces and objects in the environment. Infants, in turn, may attend preferentially to speaking faces resulting in face processing biases. We also argue that perceptual narrowing/tuning is likely the result of multiple interacting factors and not explained by the development of a single mechanism. Future work that carefully examines bottom-up and top-down influences and uses multiple methods or levels of analysis will better elucidate the behavioral and neural factors involved in perceptual narrowing and tuning for speech and faces.
